# Setting research priorities across science, technology, and health sectors: the Tanzania experience

**DOI:** 10.1186/s12961-015-0002-2

**Published:** 2015-03-12

**Authors:** Sylvia de Haan, Rose Kingamkono, Neema Tindamanyire, Hassan Mshinda, Harun Makandi, Flora Tibazarwa, Bruno Kubata, Gabriela Montorzi

**Affiliations:** The Council on Health Research for Development (COHRED), Route de Morillons 1-4, 1211 Geneva, Switzerland; The Tanzania Commission for Science and Technology (COSTECH), PO Box 4302, Ali Hassan Mwinyi Road, Kijitonyama, Dar es Salaam Tanzania; The NEPAD Agency, PO Box 1234, Halfway House, Midrand, South Africa

**Keywords:** Governance, Priority setting, Research systems, Science and technology, Tanzania

## Abstract

**Background:**

Identifying research priorities is key to innovation and economic growth, since it informs decision makers on effectively targeting issues that have the greatest potential public benefit. As such, the process of setting research priorities is of pivotal importance for favouring the science, technology, and innovation (STI)-driven development of low- and middle-income countries.

**Methods:**

We report herein on a major cross-sectoral nationwide research priority setting effort recently carried out in Tanzania by the Tanzania Commission for Science and Technology (COSTECH) in partnership with the Council on Health Research for Development (COHRED) and the NEPAD Agency. The first of its type in the country, the process brought together stakeholders from 42 sub-sectors in science, technology, and health. The cross-sectoral research priority setting process consisted of a ‘training-of-trainers’ workshop, a demonstration workshop, and seven priority setting workshops delivered to representatives from public and private research and development institutions, universities, non-governmental organizations, and other agencies affiliated to COSTECH.

**Results:**

The workshops resulted in ranked listings of research priorities for each sub-sector, totalling approximately 800 priorities. This large number was significantly reduced by an expert panel in order to build a manageable instrument aligned to national development plans that could be used to guide research investments.

**Conclusions:**

The Tanzania experience is an instructive example of the challenges and issues to be faced in when attempting to identify research priority areas and setting an STI research agenda in low- and middle-income countries. As countries increase their investment in research, it is essential to increase investment in research management and governance as well, a key and much needed capacity for countries to make proper use of research investments.

**Electronic supplementary material:**

The online version of this article (doi:10.1186/s12961-015-0002-2) contains supplementary material, which is available to authorized users.

## Background

National research and innovation policies, priorities, and management constitute the three foundations of a research and innovation system. While the policies define the aims and values that guide national research and innovation development, the priorities inform on the key research and innovation areas where the country should focus its investment, while the management plan provides the operational framework necessary to ensure coherence between policies, priorities, and action.

Clearly defined national research priorities are essential to guide research expenditure, to promote science, technology, and innovation (STI), to stimulate human resource development for research, and to inform negotiation processes with external partners for targeted funding and long-term efforts [1].

The African Union pledged that all countries in the region would aim to spend 1% of their gross domestic product (GDP) on Research and Development (R&D). In 2010, a survey conducted by the African Union among 19 countries in the region found that only three countries – Malawi, South Africa, and Uganda – met this 1% spending goal, while R&D spending in the other 16 countries ranged between 0.2% and 0.48% [2].

The Government of Tanzania is engaged to optimize R&D capacity in the country as a means to accelerate economic development, and although Tanzania has not yet managed to achieve the 1% goal, steps are actively being taken to fulfil this commitment by 2015. In 2005, Tanzanian President, Benjamin Mkapa, announced the commitment to spend 1% of the country’s GDP on R&D by 2015, a major increase from the 0.3% of Tanzania’s GDP expenditure for R&D at that time [3]. This pledge was upheld by the incumbent President, Jakaya Kikwete [4]. By 2014, expenditure on R&D is estimated at 0.52% [5]. This still falls short on the 1% target, but it does show a significant increase over the past years. In 2010, Tanzania issued its latest National R&D Policy [6], outlining the need for setting up and periodically reviewing research priority areas, and for identifying strategic research areas that address national problems and contribute to socio-economic development.

The Tanzania Commission for Science and Technology (COSTECH) was established in 1986 by the Act of Parliament No. 7 as the principal advisory organ to the Government on all matters pertaining to scientific research, technological development, and coordination of research activities in the country. Through consultation and cooperation, COSTECH brings together the nation’s scientific and technological institutions to promote, coordinate, monitor, and evaluate scientific research and innovation. COSTECH manages and administers loans and grants to institutions and researchers engaged in research that contribute to the development of STI and that are in compliance with the Government’s national priorities [7].

In order to respond to the National R&D Policy of 2010 and to the improved Tanzania R&D financial context, it was essential for COSTECH to develop a solid research agenda that would guide the fair allocation of new resources across all sectors. To this end, COSTECH partnered with the Council on Health Research for Development (COHRED) and the NEPAD Agency (through the Research for Health Africa programme) in February 2011 to implement the first cross-sectoral nation-wide research priority setting effort in Tanzania, bringing together the science, technology, and health sectors. This constituted the first phase of a long-term priority setting process led by COSTECH, implemented between April and August 2011. Following the process on the mainland, COSTECH supported a separate priority setting process in Zanzibar during 2013. This article is the first in a series of two, and reports on the priority setting process conducted on the mainland. The second article will include the results from Zanzibar, which followed a different process, and will focus on the implementation aspects of agenda setting.

## Methods

### Choosing the priority setting method

Countries that have conducted priority setting processes have employed a range of methods from those developed by the countries themselves, to Delphi-like procedures, to those methods which have been externally developed and tested, including the Essential National Health Research Approach, the Combined Matrix Approach, the Advisory Committee Approach, the Ad Hoc Committee Approach, and the Child Health and Nutrition Research Initiative Approach, among others [8-11].

For COSTECH, the key decision points for choosing a method were:

- The process should be carried out within a very short timeframe (April – August 2011);
- It should involve all sectors;
- Consensus building among stakeholders was given high importance;
- The process would have to be expert driven, as there were no financial resources for a situational analysis of research conducted in the past (nor was this information readily available); and
- The research agenda should be set for a period of 3 to 5 years, with a mid-term review to accommodate emerging priority issues.

Based on these contextual realities, we opted for defining priorities through consultative expert workshops that would be guided by a clear process, criteria, and ranking framework. It was considered feasible, within the given timeframe and budget, to organize eight consultative expert workshops that would cover a total number of 42 sub-sectors. Due to time and budget limitations it was not possible to hold a workshop per sub-sector, nor was this considered advisable as many research issues crosscut a range of sectors.

**Figure 1:**
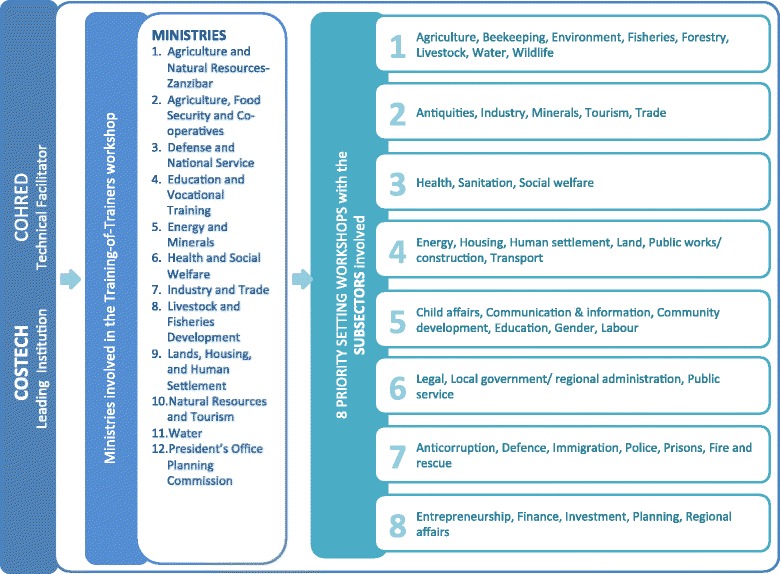
**Schematic representation of the cross-sectoral priority setting process implemented in Tanzania.**

To ensure coherence across these workshops, it was decided to start off with a training-of-trainers workshop, training the facilitators of the eight planned sectoral workshops. The priority setting process is schematically summarized in Figure 1.

### Defining the stakeholders

The institutions affiliated with COSTECH include both public and private research and development institutions, across all sectors, as well as institutions of higher learning and non-governmental organizations (NGOs). The public institutions operate under their respective ministries. COSTECH’s approach to identifying the stakeholders focused on these ministries.

For the training-of-trainers workshop, the following ministries were approached and were asked to nominate two people that would afterwards be responsible for facilitating their sectoral workshops:

- Ministry of Agriculture and Natural Resources – Zanzibar
- Ministry of Agriculture Food Security and Co-operatives
- Ministry of Defence and National Service
- Ministry of Education and Vocational Training
- Ministry of Energy and Minerals
- Ministry of Health and Social Welfare
- Ministry of Industry and Trade
- Ministry of Livestock and Fisheries Development
- Ministry of Lands, Housing, and Human Settlement
- Ministry of Natural Resources and Tourism
- Ministry of Water
- President’s Office, Planning Commission

From these 12 governmental bodies, the Ministry of Agriculture and Natural Resources in Zanzibar and the President’s Office Planning Commission did not have their own sectoral workshops. The Zanzibar Ministry of Agriculture and Natural Resources participated a year later in the priority setting process initiated by COSTECH in Zanzibar. The President’s Office Planning Commission participated as observer to the process. Four other ministries joined forces and organized two sectoral workshops: the Ministry of Agriculture, Food Security, and Cooperatives held their workshop jointly with the Ministry of Livestock and Fisheries Development. The Ministry of Energy and Minerals held their workshop jointly with the Ministry of Lands, Housing, and Human Settlement. Therefore, although 12 ministerial bodies participated in the training-of-trainers workshop, only eight sectoral workshops were held.

For the sectoral workshops, COSTECH sent letters to the permanent secretaries or the head of ministries, and to heads of university departments, asking to nominate experts to attend the sectoral workshop. For each sub-sector attending a sectoral workshop a maximum of 10 participants were invited. The aim was to obtain a good representation from across the relevant institutions (including government bodies, research institutions, and NGOs) of a sub-sector. The participants of the training-of-trainers workshop were also used to follow-up on the nomination process.

### Priority setting criteria

Throughout the priority setting process three different ‘filters’ were used to prioritize the areas defined: i) The first filter, applied during the second day of the sectoral workshops, focused on the research itself and asked participants to address the following questions: What is the potential for research utilization of the proposed research area? Would the research area involve the development of products or have the potential to improve services? Would the proposed research area bring an innovative element? Would it enhance entrepreneurship?; ii) The second filter, used during the third day of the sectoral workshops, emphasized the dimension of relevance and the opportunity for cross-sectoral work (see Table 1 for a list of the criteria used for ranking of research priority areas); and iii) The third filter, applied during the narrowing process which followed the sectoral workshops, added the dimension of market potential and thus focused more on development than research (see below for a list of criteria used for producing final list of research priorities).

**Table 1:** **Criteria for ranking research priority areas**

**Criteria**	**As determined by**
Appropriateness	Ethical and moral issues
	Availability of pre-existing data
	Culturally accepted
Relevance	Equity focus and community concern/demand
	The size of the problem
	Contributes to the national and sector objectives
Feasibility	Capacity of the system to support the research
	Financial and human resources available
	Cultural/political environment
Impact of research outcome	Chance/opportunity to implement the research
	Use of the research results
	Link of the research to policy decisions
	Overall reduction of the problem, including cost
Opportunity to strengthen collaboration with partners	Presence of capable partners
	Availability of partner infrastructure and resources
	Possibility that potential partners will collaborate to undertake the research
	Possibility of greater research outcome with partner involvement

#### Criteria for producing final list of research priorities

i) Linkage to the National Five Year Development Plan; ii) feasibility for implementing the research; and iii) the possibility for cross-sectoral work. The Tanzania Five Year Development Plan covers 2011/2012 to 2015/2016 as a means to implement the Tanzania development vision 2025. It is the first in a series of three Five Year Development Plans, which aim to transform Tanzania into a middle-income country by 2025.

### Training-of-trainers workshop

A two-day training-of-trainers workshop, in which 21 experts participated, was held in April 2011 in Arusha, Tanzania. The trainers consisted of COSTECH staff members jointly with COHRED technical experts. Participants were senior representatives from affiliated ministries and institutions.

In preparation for the workshop, an 18-page facilitator guide was developed. This guide was used throughout the workshop to familiarize the participants with the design, technical components, and standardized tools that would be used in the eight priority setting workshops to be rolled out to the different sub-sectors. The facilitator guide addressed every issue from sectoral workshop start to finish: it presented a draft agenda for the sectoral workshops, outlined the detailed process to be followed, and gave facilitator tips for dealing with potential conflicts during the workshop. The aim was to ensure future facilitators understood and were confident with their facilitator role, and that they would be able to use the facilitators guide in their sectoral workshops, thus ensuring a maximum level of consistency between the eight sectoral workshops.

The training workshop led to one change in the methodology proposed for the sectoral workshops. Participants added one additional criteria against which research areas would be rated during the last day of the prioritization phase in each priority setting workshop. Four criteria (appropriateness, relevance, feasibility, and impact of research outcome) were derived from the Essential National Health Research Strategy approach and were proposed by the trainers [12]. One further criterion, ‘Opportunity to strengthen collaboration with partners’ was proposed by the workshop participants as it was considered relevant to COSTECH’s role in strengthening collaboration and partnership between the sectors and institutions it collaborates with.

Participants discussed the desired breadth of the priority setting exercise. Should the aim be to define broad research areas or more clearly defined research topics? While defining research topics would result in a more operational research agenda, participants agreed that it might not be achievable in a first nationwide priority setting process to get to the level of detail needed to define priority topics. It was therefore agreed to define research areas. It was also clearly stated that going to the level of research questions was not desired, as researchers would eventually propose research questions within the scope of the proposals they will submit to address the priority research areas defined.

Another discussion took place around the possibility of obtaining tied ranking when going through the ranking process. It was agreed to see tied scoring results as research areas that were given equal priority.

Given the lack of background of participants in the area of priority setting and also due to time limitations, the focus of the training-of-trainers workshop was on the theoretical elements associated with priority setting. Only little time was available for practical training in facilitation processes. The group therefore decided to treat the first sectoral priority-setting workshop as a demonstration workshop, with involvement of all the trainers from the training-of-trainers group. This would give them the opportunity to gain practical facilitation experience prior to the implementation and their involvement in the seven remaining workshops.

Following the training-of-trainers workshop, changes were made to the facilitator’s guide, which was disseminated to all participants of the training workshop.

### Demonstration workshop

From May 18–20, 2011, the first sectoral workshop was held. The demonstration workshop involved participation from eight sub-sector groups covering agriculture, beekeeping, environment, fisheries, forestry, livestock, water, and wildlife. COSTECH sent letters to the relevant affiliated ministry institutions, universities, and NGOs, asking that participants be identified. COSTECH also requested that the selected participants bring any relevant information and materials regarding past research conducted, current research underway, and future research planned. If the ministry or institution had previously engaged in priority setting either within the sector group or within the single institution, COSTECH requested that this information be available at the priority setting workshop.

Over 60 participants attended the demonstration workshop, including 15 participants from the training-of-trainers workshop. The representatives from the training-of-trainers workshop met with COSTECH staff and workshop facilitators a day prior to the three-day workshop to address process issues and identify their roles in specific workshop sessions.

The three-day workshop had the following structure:

*Day 1: Setting the stage – what do we know?*

Presentations focused on providing an overview of current research, major research areas, questions to be addressed, research collaborations, and available resources. Any data available from information systems demonstrating the degree of current problems were used. Day 1 resulted in a list of (maximum 50) outstanding problems and questions, as well as a list of (maximum 50) research areas presented.

*Day 2: Research topics important to decision-makers and researchers*

At the start of day 2, participants received copies of the lists produced on the first day with outstanding research problems, questions, and areas. In addition, the facilitator provided a summary of the first day. Following this overview, participants were divided into small groups of 5 to 8 experts representing various institutions of a sub-sector. The small groups were given the task to identify areas, from the lists provided, that are already researched and that do not need further research. The groups reported back to the plenary following their discussions. Only areas on which consensus existed were included in the list of areas not needing further research.

Following this first exercise of excluding areas not needing further research, the small groups were given the task to list a maximum of 20 priority research areas. The groups were asked to consider four key questions when discussing the priority areas (applying the first priority setting filter defined above).

The groups reported back to the plenary following their discussions. During this process a facilitator kept track of areas that were listed across several groups. The expectation was that there would be considerable overlap in priority areas identified, or that it would be possible to combine areas from the various groups. The aim was to have, at the end of day 2, a consolidated list of maximum 20 research areas per sub-sector representing the needs of both researchers and decision-makers.

*Day 3: Rating and ranking of the research priorities*

Day 3 started with a presentation on the rating process whereby participants were taught how to perform the individual and group rating activities. Following the presentation, each participant was asked to conduct an individual rating of the research areas identified, using the criteria of Table 1 (thus applying the second priority setting filter) and giving each criteria a rate of 1 to 5, 5 being the highest. Prior to the individual rating process, the chairperson of each small group made sure that the participants were well conversant with the research areas proposed. This allowed for questions of clarification, facilitating the rating process, and avoiding misunderstanding and potential mis-rating. However, it was important that the chairperson not allow further discussion about the research areas listed on day 2, which could influence the rating process. After individual rating, the results of each participant were compiled in a group score sheet, followed by compiling the scores per research area in an overall rating sheet where the ranking was done. This process was followed by a discussion within the small group on areas where there was discrepancy among the members. However, a change in rating was not allowed. The small groups presented their results in the plenary. The final plenary of the workshop focused on assessing the consensus of people around the ranked research areas. This also helped identify any outstanding issues, considerations, or concerns for COSTECH to take on board and reflect on how the priority setting process can remain fair and transparent.

The demonstration workshop provided the first opportunity to pilot the facilitator’s guide. The facilitator’s guide, workshop sessions, including the length of these sessions, and the tools that had been developed for use in each session were evaluated during the course of the demonstration workshop to determine whether any final changes needed to be made prior to implementing the remaining seven workshops. No changes were deemed necessary for the remaining seven sectoral workshops.

### Priority setting workshops

Following the demonstration workshop, seven other sectoral priority-setting workshops were held (Table 2). These workshops followed the same process and methodology as described above for the demonstration workshop.

**Table 2:** **Dates, participating sub-sectors, and participants of the sectoral workshops**

**Number**	**Date**	**Participating sectors**	**Total number of participants**	**Participants per stakeholder group (in %)**				
				**Universities**	**R&D institutions**	**Governmental departments**	**Private sector**	**Civil society**
1*	May 18–20, 2011	Agriculture	53	13%	24%	53%	6%	4%
		Beekeeping						
		Environment						
		Fisheries						
		Forestry						
		Livestock						
		Water						
		Wildlife						
2	June 1–3, 2011	Antiquities	30	13%	30%	44%	10%	3%
		Industry						
		Minerals						
		Tourism						
		Trade						
3	June 15–17,2011	Health	20	25%	35%	20%	10%	10%
		Sanitation						
		Social Welfare						
4	June 29–July 1, 2011	Energy	43	21%	23%	54%	0%	2%
		Housing						
		Human settlement						
		Land						
		Public works/construction						
		Transport						
5	July 13–15, 2011	Child affairs	47	24%	4%	53%	13%	6%
		Communication & information						
		Community development						
		Education						
		Gender						
		Labour						
6	July 27–29, 2011	Legal	26	4%	15%	77%	0%	4%
		Local government						
		Regional administration						
		Public service						
7	August 10–12, 2011	Anti-corruption	40	0%	12%	80%	0%	8%
		Defence						
		Immigration						
		Police						
		Prisons						
		Fire and rescue						
8	August 24–26, 2011	Entrepreneurship	43	14%	23%	49%	14%	0%
		Finance						
		Investment						
		Planning						
		Regional affairs						

*Demonstration workshop.

### Narrowing research areas

In the planning phase of the priority setting process, it was expected that the eight sectoral workshops would be able to develop an overall agenda cutting across the sub-sectors that participated. During the training-of-trainers workshop it became clear that participants were not ready to define research priorities across the participating sectors. Instead of developing one priority-listing cutting across the sub-sectors, the participants opted for developing a research agenda for each of the sub-sector participating. At the end of the 4 months workshop period, COSTECH had thus a listing of research priorities for each sub-sector, totalling approximately 800 priorities across the 42 sub-sectors. In view of the extensive list of priorities identified, COSTECH appointed an expert panel to assess the 800 research priorities against the three criteria indicated above (the third priority setting filter, criteria for producing final list of research priorities) with the aim of reducing the list to a manageable instrument that could be used to guide research investment. The criteria chosen were different from the criteria used during the ranking workshops because, at the time the expert panel met, the National Five Year Development Plan had just been released in draft version, which allowed COSTECH to align itself to this plan. In addition, the aim of the narrowing process was to support COSTECH in allocating increased financial resources in such a way that it could show impact of this investment in the medium term. It was thus essential that proposed research would be feasible, and that it supported cross-sectoral work in the best possible way.

The expert panel met on 22 Sept, 2011, and on 5 and 6 October, 2011, and was composed of COSTECH (as process leader), the Planning Commission (as the organization in charge of, among other, developing Tanzania’s Five Year Development Plan), Policy Research for Development (as an NGO conducting poverty research studies), and the National Institute for Medical Research (as the organization with most experience in research priority setting processes in Tanzania).

In first instance, the expert panel reviewed whether the sub-sectors were mentioned in the Tanzania Five Year Development Plan. Four sub-sectors (anti-corruption, immigration, fire and rescue, and regional affairs) were not included in this plan and were thus excluded from the final review of research priorities. For the remaining 38 sub-sectors the panel assessed whether there were any research areas defined within the eight workshops that were overlapping in nature and could thus be combined. The panel also assessed whether certain items were programmatically focused as opposed to being research focused, and removed the programmatic priority areas. The latter illustrates the challenges that arose in some of the workshops, especially those involving sectors that had never before defined research priorities, in distinguishing between what needs research to solve and what are priority issues to be addressed through better programme management, for example. Research areas that the expert panel did not consider as relevant to the National Five Year Development Plan, were not considered feasible, or not supporting cross-sectoral work, were excluded from the priority list. This narrowing process resulted in a reduction of the priority list from over 800 to 140 research areas.

## Results

The eight sectoral workshops organized between May and August 2011 resulted in a total list of over 800 priority areas for 42 sub-sectors (see Additional file 1 for the complete ranked list of research priorities). For management purposes and for allocating financial resources in priority areas, this list was not practical. COSTECH thus decided to narrow the list down using the expert panel as described in the methods section.

The expert panel separated the priorities into four main categories (Additional file 1): productive sector, services sector, economy, and governance, with each category specifying research priorities by sub-sector. This process resulted in a reduced list of 140 research areas, 54 research areas for the productive sector, 60 for the services sector, 15 in the category of economy, and 11 research areas focusing on governance issues. As noted in Additional file 1, not all sub-sectors have the same number of priority research areas. The sectoral workshops allowed a maximum of 20 research areas to be defined per sub-sector. Some sub-sectors, however, decided to prioritize fewer research areas. In addition, the narrowing process allowed merging of similar ideas across sub-sectors leading to varying numbers of priority areas per sub-sector.

Due to the process of reducing and combining research areas across sectors, the original ranking of the research areas was distorted. Therefore, the final priority research areas do not present a hierarchy or ranking. In addition to these priority research areas, the expert panel proposed the issues of policy, governance, and human rights to be considered throughout as these issues appeared on many of the sub-sector’s agendas.

## Discussion

### Relevance of priority setting

For many developing countries, conducting research and addressing health needs is often dependent on or guided by external resources coming into a country rather than based on the needs identified by the countries themselves, which results in diverting attention and resources away from national or sub-national interests [13].

It is through the creation of a national research agenda that countries can begin to align both external and internal resources to best meet national development and equity needs and improve people’s livelihood. National research priority setting i) facilitates the transformation of a donor-driven research agenda into an agenda driven by countries’ own needs [8]; ii) highlights the investment in research in a fair and legitimate way, using a sound and transparent methodology [9]; iii) maximizes the impact of investments, which is especially critical to resource-poor environments, through the allocation of funding into research areas of strategic importance [3,14]; iv) through the use of clear criteria and principles, it guides investments based on a vision of what the endpoints of such investments should be [10]; v) provides the foundational elements for building a strong national research governance system based upon the synergistic needs defined by a country’s stakeholders; vi) countries that have engaged in priority setting have provided their decision makers with solid foundations for negotiating with donor agencies to support national research [15].

A national research priority setting process is necessarily shaped by the country’s current reality; therefore, there are no specific recommendations on which approach or tool would be best to use [2,16]. There is, however, agreement on key principles defining the development of a sound priority setting process.

#### Including broad representation of all stakeholder groups, so that each group’s voice contributes the priorities identified through the process

Active involvement of the stakeholder groups helps create a sense of ‘ownership’ in the process and add much value to the research priorities identified as a result of such processes [12]. An inclusive priority setting process will help ensure that [17,18] i) important research topics and areas are not overlooked; ii) identified priority research is implemented, because the stakeholders themselves have selected research needs and acquired a sense of ownership over them; iii) priorities are a better match to societal and policy needs of the country; iv) duplication of research efforts and the resulting waste of precious resources are avoided; and v) there is shared responsibility for implementing the national research agenda.

#### Ensuring a systematic and transparent process and not losing sight of the fundamental questions*:* whose voices are heard, whose views prevail and, thus, whose interests are advanced [1,19]

Given the diversity of stakeholder groups participating in a priority setting effort, and their different perspectives, e.g., medical, public health, economical, social, legal, political perspectives, the effort needs to consider how any potential conflicts between the various perspectives will be addressed [6].

#### Guaranteeing relevancy by periodic review and updating

Research priority setting involves a continuous process which requires coordination and periodic re-evaluation with feedback from the previous efforts for continually improving the process [3,13], to address emerging and shifting health and development issues.

For COSTECH and Tanzania the relevance of this first nationwide cross-sectoral priority setting process is observed in various developments that followed the process:

- The agenda has guided a call for proposals issued by COSTECH in 2014;
- The process helped strengthen capacity of COSTECH staff, as well as stakeholders who participated in the various workshops, in using priority setting approaches in a cross sectoral and multi-disciplinary environment. This is, for example, illustrated by research institutions in the area of Infrastructure and Information and Communication Technology that have used the methodology to generate their own priority research areas;
- The developed approaches and tools have been modified and been applied by COSTECH to undertake a similar exercise in Zanzibar;
- The results of the priority setting process have been shared at various forums, workshops, and seminars.

### Lessons learnt from cross-sectoral priority setting in Tanzania

The priority setting effort conducted by COSTECH was an immense undertaking, involving 42 sub-sectors within the fields of STI. Implementing eight workshops covering all these sub-sectors in less than 4 months was a major challenge. This was the first priority setting effort of such scope and nature in Tanzania, thus presenting a number of challenges along its implementation way.

#### *S*takeholders’ inclusiveness

Given the broad constituent base covering such a large number of sub-sectors, costs were prohibitive for ensuring inclusiveness across sub-sectors. Sub-sector representation was quite small in some instances, ranging anywhere from two to eleven individuals representing a specific sub-sector. This raises the concern on how balanced and equitable the contribution of each sub-sector was. A possible way of adjusting future priority setting efforts of this nature would be to set minimum criteria for what could be considered an equitably inclusive process.

#### Training-of-trainers workshop

The training-of-trainers workshop proved to be a useful strategy to train people for their roles during the sectoral workshops. The trainers were of great help during the small group discussions of their respective sectoral workshops. Two trainers felt comfortable chairing and facilitating their entire sectoral workshop. For the other sectoral workshops, COSTECH staff took on the main facilitating role. Although the facilitators would probably have been capable of facilitating the workshops, they felt it was COSTECH responsibility to lead the process, with their assistance. Nevertheless, COSTECH staff would not have managed to facilitate all workshops and group work without the support of the trained facilitators.

#### Process standardisation

Setting a common baseline for all sub-sectors along the priority setting process was very difficult, primarily because the sub-sectors were at different stages of developing their own research agenda. Some sub-sectors, i.e., health, agriculture, wildlife, environment, fisheries, and forestry, were quite advanced and built on previous processes, using this previous information as an input for the STI research agenda. Other sub-sectors were much less advanced, struggled with the priority setting process, which was a new experience to them, and did not have much information to feed into the process. This raises the concern of whether process standardization is something that COSTECH should actually aim for. An alternative approach would be to stimulate and guide sub-sectors into setting their own research agenda, and subsequently bring the sub-sectors’ agendas together to identify which are the areas that most closely align with national development plans and that COSTECH can support. This approach has the potential to increase the sub-sector’s agenda ownership, which could lead to a more proactive attitude from sub-sectors to mobilize funds for financing their own priorities, when these cannot be supported by COSTECH.

#### Alignment with national efforts

The cost-effectiveness of a priority setting process is highly dependent on its timeliness. In Tanzania, the process occurred parallel to the redefinition of the national development plan. It is only when 800 research priorities where identified that a small expert panel tried to align these priorities with the National Five Year Development Plan, which became only then available as draft. The key to a successful priority setting process lies in its alignment with major policies, strategies, and development plans. In future, such alignment should be contemplated in the planning phase of the priority setting effort rather that in the implementation phase.

#### Specificity of research priorities

A cross-sectoral priority setting process, like the one described herein, inevitably leads to a broad STI research agenda that has the value of providing strategic guidance. However, under this umbrella, more specific research agendas are required at sector level for management purposes.

#### Using existing priority setting methods

Prior to starting the priority setting process, COSTECH staff reviewed a series of priority setting methods, especially from the field of agriculture. They felt, however, that their specific contextual factors, most notably time pressure, limited financial resources, and needs for inclusiveness, required an adaptation of existing processes and methods specifically tailored to the needs of COSTECH. Due to these factors it was, for example, not possible to conduct in-depth situation analysis for each of the sectors prior to embarking on the priority setting process. In discussion with the technical team from COHRED, a process was thus designed for COSTECH that would allow the defining of a cross-sectoral research agenda for the first time. While realizing the process would not be perfect, COSTECH management decided it would aim to improve the process in future revision rounds. In any priority setting process, different contextual factors will determine what methods and processes are feasible and it may be difficult to apply an existing method in a given context without modification.

## Conclusions

By engaging in this challenging effort, COSTECH has made possible the development of a first STI research agenda for Tanzania that will constitute the basis of subsequent improved efforts. Similarly to COSTECH, the National Institute for Medical Research engaged in a long term priority setting process several years ago, and has recently completed its fourth priority setting cycle. Each cycle has offered the opportunity to gain more experience and improve the process, thus resulting, every time, in a better, more valuable instrument for guiding national investment in research.

As countries increase their investment in research, it is essential to increase investment in research management and governance as well, a key and much needed capacity for countries to make proper use of research investments.

## Additional file

Additional file 1:
**Ranked priorities defined through the sectoral workshops.**

## Abbreviations

COSTECH: Tanzania Commission for Science and Technology
COHRED: Council on Health Research for Development
GDP: Gross domestic product
NGOs: Non-governmental organizations
R&D: Research and Development
STI: Science, technology, and innovation
